# Digestive system in psoriasis: an update

**DOI:** 10.1007/s00403-017-1775-7

**Published:** 2017-09-13

**Authors:** Daniel Pietrzak, Aldona Pietrzak, Dorota Krasowska, Andrzej Borzęcki, Kinga Franciszkiewicz-Pietrzak, Beata Polkowska-Pruszyńska, Maja Baranowska, Kristian Reich

**Affiliations:** 1 Department of Anaesthesiology and Intensive Care, Medical University of Lublin, Lublin, Poland; 2 Chair and Department of Dermatology, Venereology and Paediatric Dermatology, Medical University of Lublin, Lublin, Poland; 3 Chair and Department of Hygiene, Medical University of Lublin, Lublin, Poland; 4 Department of Surgical Oncology, Medical University of Lublin, Lublin, Poland; 5 Student’s Scientific Association at the Department of Dermatology, Venereology and Pediatric Dermatology, Medical University of Lublin, Lublin, Poland; 6 Institute of Fine Arts University of Warmia and Mazury in Olsztyn, Olsztyn, Poland; 7 Dermatologikum Hamburg, Hamburg, Germany

**Keywords:** Psoriasis, Oral cavity, Geographic tongue, Inflammatory bowel disease, Celiac disease, Non-alcoholic fatty liver disease, Cancer

## Abstract

Psoriasis is a chronic inflammatory immune-mediated disorder associated and often coexisting with many other immune-related clinical conditions including those affecting the gastrointestinal tract. Data obtained from the reviewed literature suggest an association between psoriasis and pathologies of the oral cavity, both psoriasis-specific lesions, as well as non-specific, such as geographic tongue or fissured tongue. These findings show the importance of thorough examination of oral mucosa in psoriatic patients. Inflammatory bowel diseases (IBD) are also linked with psoriasis. Crohn’s disease and ulcerative colitis share a common genetic background, inflammatory pathways and have an evident iatrogenic anti-TNF treatment link, necessitating dermatological or gastroenterological care in patients with IBD or psoriasis, respectively, as well as treatment adjusted to manifestations. The presence of celiac disease-specific antibodies in psoriatic patients and their correlation with the severity of the disease show the association between these disorders. The linking pathogenesis comprises vitamin D deficiency, immune pathway, genetic background and increase in the intestinal permeability, which suggests a potential benefit from gluten-free diet among psoriatic patients. The link between psoriasis and non-alcoholic fatty liver disease implies screening patients for components of metabolic syndrome and lifestyle changes necessity. Some studies indicate increased prevalence of cancer in patients with psoriasis, probably due to negative influence of skin lesion impact on lifestyle rather than the role of psoriasis in carcinogenesis. However, there are no sufficient data to exclude such an oncogenic hit, which is yet to be confirmed. Therefore, all psoriasis-associated comorbidities establish the importance of a multidisciplinary approach in the treatment of these patients.

## Introduction

Research evidence from the recent decade suggests that psoriasis, a chronic inflammatory immune-mediated disorder affecting ca. 2–11.4% of individuals from developed countries [125], is not an isolated pathology of the skin, but a systemic condition involving multiple organs and systems [58]. Moreover, due to similarity of pathogenic pathways, psoriasis may predispose, or at least coexist, with other genetically determined immune-mediated chronic inflammatory conditions [125]. While association of psoriasis with psoriatic arthritis and conditions forming the so-called metabolic syndrome (i.e., central obesity, insulin resistance, hypercholesterolemia, atherosclerosis, arterial hypertension, cardiovascular diseases) is well established, also a number of other entities that occur more often in psoriatic patients have been identified recently, among them inflammatory bowel disease (IBD), celiac disease, non-alcoholic fatty liver disease (NAFLD), uveitis, osteoporosis and depressive disorders [65] (Tables 1, 2). While it is still unclear if psoriasis is a predisposing factor or rather a consequence of these conditions, available evidence suggests that their coexistence is not random. This makes these findings vitally important from a diagnostic and therapeutic perspective.

**Table 1:** Immunometabolic components of psoriatic process include arterial hypertension, atherosclerosis, cardiovascular diseases, central obesity, dyslipidemias, insulin resistance and metabolic syndrome (in alphabetical order)

Immunometabolic components of psoriatic process
Arterial hypertension
Atherosclerosis
Cardiovascular diseases
Central obesity
Dyslipidemias
Insulin resistance
Metabolic syndrome

**Table 2:** Other possible components of psoriatic process include celiac disease, depressive disorders, inflammatory bowel disease (IBD), non-alcoholic fatty liver disease (NAFLD), osteoporosis, uveitis and others

Other possible components of psoriatic process
Celiac disease
Depressive disorders
Inflammatory bowel disease (IBD)
Non-alcoholic fatty liver diseases (NAFLD)
Osteoporosis
Uveitis
Others

A review of available literature suggests that a large proportion of diseases being epidemiologically linked to psoriasis involve the gastrointestinal tract (GI) [58]. Therefore, the aim of this paper is to review published data on the GI pathologies that frequently coexist with psoriasis, their effects on natural history of this condition, potential shared pathogenic mechanisms, diagnostic and therapeutic implications.

## Materials and methods

A search of Medline and EMBASE from 1966 to 2016 was carried out. The date of the last search was September 2016. The database was searched using the relevant MeSH terms including all sub-headings. The studies reporting the association between digestive system disorders and psoriasis were identified from the database by utilizing the search terms (“gastrointestinal” OR “digestive” OR “liver” OR “mouth” OR “oral cavity” OR “intestine” OR “inflammatory bowel disease” OR “Crohn’s disease” OR “ulcerative colitis” OR “celiac disease” OR “gluten intolerance” OR “non-alcoholic fatty liver disease” OR “liver steatosis” OR “cancer” OR “neoplasm” OR “geographic tongue” OR “fissured tongue” OR “comorbidity”) AND (“psoriasis” OR “psoriatic”). We searched for English-language publications and human studies. The database search revealed 5835 records. We chose the most representative (most insightful, explaining or suggesting the pathophysiology of the described conditions) studies and our article is not a meta-analysis.

## Results

### Oral cavity disorders (psoriasis-specific lesions, geographic tongue and fissured tongue)

**Fig. 1:**
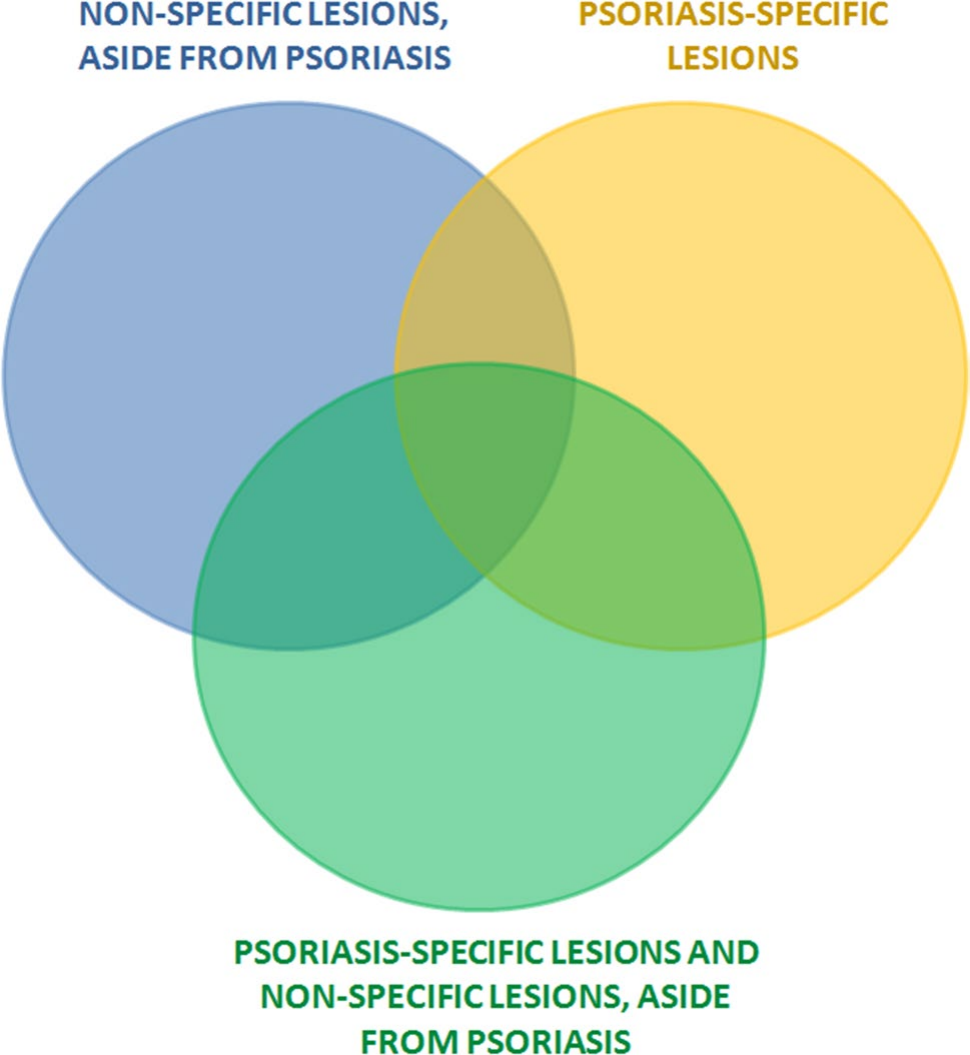
Mucous lesions found in the oral cavity of psoriatics are divided into non-specific lesions, aside from psoriasis and psoriasis-specific lesions. Based on Xing et al. [171]

Although the occurrence of oral psoriatic lesions was first described by Oppenheim already in 1903 [130], the involvement of extra-dermal tissues by psoriasis has been for many years put into question [110, 175]. It was quite recently, when the term ‘oral psoriasis’ becomes widely accepted owing to a growing body of evidence for a systemic character of this disease [175]. While psoriatic lesions can be found virtually everywhere in the oral cavity, they most commonly involve mucosal membranes of the tongue, cheeks and gums [55, 133]. Clinical presentation of oral psoriasis is highly heterogeneous which makes the diagnosis quite challenging [47, 175]. Furthermore, evaluation of the oral mucosa usually is not a routine component of a dermatological examination in psoriasis [133]; therefore, the true incidence of oral psoriasis may be underestimated. Mucous lesions found in the oral cavity of psoriatics can be divided into two groups: (1) psoriasis-specific lesions, and (2) non-specific lesions, present aside from psoriasis, observed also during the course of other conditions [41, 133] (Fig. 1).

**Fig. 2:**
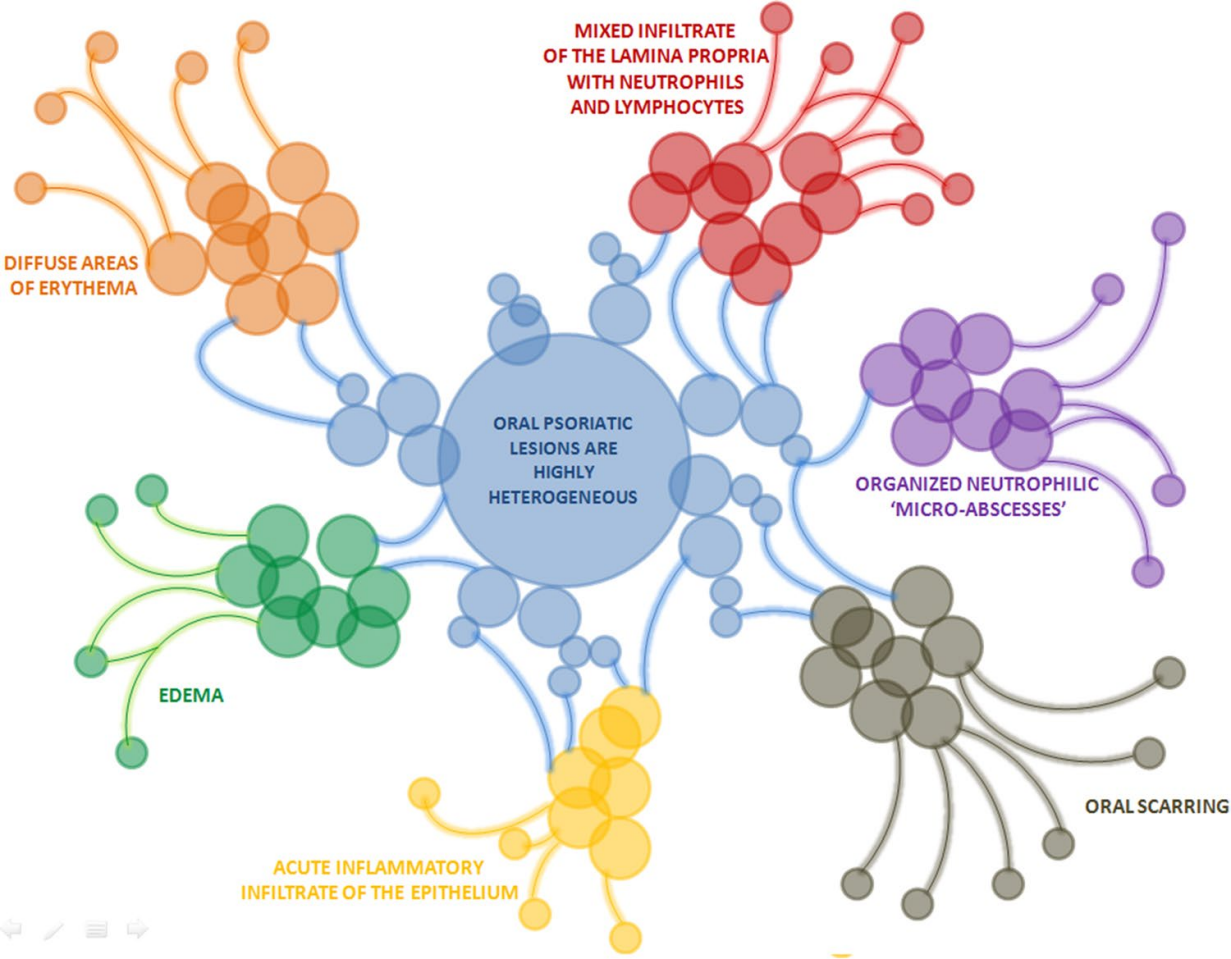
Clinical presentation of oral psoriasis is highly heterogeneous and may present as oral scarring, diffuse areas of erythema, edema, acute inflammatory infiltrate of the epithelium, mixed infiltrate of the lamina propria with neutrophils and lymphocytes and organized neutrophilic ‘micro-abscesses’. Based on Xing et al. [171]

The first group comprises mucosal lesions, which histological presentation is similar to that of skin psoriasis. Usually, these mucosal lesions coexist with involvement of the skin, but the cases in which oral psoriasis preceded development of dermal lesions have been reported as well [34]. Oral psoriatic lesions are highly heterogeneous. They may present as white or gray plaques, annular lesions, diffuse areas of erythema, edema, acute inflammatory infiltrate of the epithelium and mixed infiltrate of the lamina propria with neutrophils and lymphocytes, and organized neutrophilic ‘micro-abscesses’ [34, 47, 116] (Fig. 2). Some patients with mucosal psoriasis present with oral scarring, which could also be related to psoriasis [34]. During recent years, oral lesions that are non-specific for psoriasis, i.e., geographic tongue (GT) and fissured tongue (FT), have been gaining a growing interest from clinicians and researchers [47]. These lesions cannot be considered psoriasis specific as they do not necessarily co-exist with the skin involvement and are also observed during the course of other conditions [130]. However, a growing body of evidence from epidemiological studies, as well as the similarity of their pathogenic pathways to those involved in skin psoriasis, suggests that a link between these conditions can be stronger than previously suspected.

Geographic tongue, described for the first time by Reiter in 1831, is a chronic inflammatory lesion of unexplained etiology [64, 77, 84, 180]. GT is found in 0.6–4.8% of individuals in the general population, more often in children and slightly more frequently in women than in men; the incidence of GT decreases with age [10, 85, 117]. The condition is characterized by serpiginous white areas around the depapillated mucosa of the dorsum and sides of the tongue [127]. The white border consists of filiform papillae in regeneration and of a mixture of keratin and neutrophils, while erythematous area results from the loss of these papillae [128]. Due to its dynamic character, periodic exacerbation and remission, disappearance of lesions in one location and their simultaneous development in another, variable pattern, size and colour, GT is also referred to as benign migratory glossitis or wandering rash of the tongue [127]. The migratory character of the lesions is associated with epithelial desquamation in one location and simultaneous proliferation in another [64]. While GT is usually asymptomatic, some patients report pain or a burning sensation, especially during consumption of spicy or acidic foods [64, 77].

Although GT is most commonly observed in psoriasis, its coexistence with diabetes mellitus, hormonal disorders, reactive bronchitis, asthma, eczema, hay fever, atopy, elevated serum IgE, other chronic inflammatory conditions, bacterial infections, anemia, dietary deficiencies, such as vitamin B complex and iron deficiency, Reiter syndrome and Down syndrome has been reported as well [18, 182]. Further, GT can be found in some pregnant women, individuals exposed to stress and some medications, including oral contraceptives and lithium salts [5, 64, 84, 117]. Also mechanical factors, e.g., talking and chewing, may stimulate development of these lesions; this is referred to as the so-called Koebner phenomenon [128]. Nevertheless, a plethora of epidemiological studies showed that GT is most common in psoriatics [75]. The incidence of GT in psoriatic patients is estimated at 5.6–18.1% [128]. GT was shown to be associated with an array of clinical characteristics of psoriasis, especially its severity [37, 75, 150, 182]. It is typically found in younger persons and seems to be a typical feature of early-onset psoriasis [182]. Early onset psoriasis is usually considered to be more severe and more strongly associated with ungual and facial involvement as compared to the late-onset disease [178]. Consequently, GT may indirectly reflect the severity of psoriasis. Furthermore, a few studies found an association between the occurrence of GT and the severity of psoriasis expressed with PASI scores [37, 150]. Moreover, Pogrel and Cram [131] suggested that patients with acute exacerbation of psoriasis develop more oral lesions than individuals with the stable disease. Finally, according to some authors, GT is more common in subjects with generalized pustular psoriasis, i.e., more aggressive form of the disease [37, 130]. Aside from being a marker of psoriasis severity, GT seems to be also a predictor of this condition. In some published case reports, the presence of GT preceded development of skin psoriasis, especially in patients with pustular disease [37]. Interestingly, histological studies demonstrated that even in non-psoriatic patients with GT, the same histopathological features of psoriasis are present in most cases [48].

As already mentioned, a growing body of evidence points not only to epidemiological but also to a causal link between psoriasis and GT. According to some authors, GT may present similar genetic, histopathological and clinical features [48, 178]. Both skin psoriatic lesions and GT have similar histopathologic presentation: regular increase of the spinous layer with thickening of the lower portions, thickening and swelling of papillae, suprapapillary hypotrophy with occasional presence of small spongiform pustules, absence of granular layer, parakeratosis, presence of Munro’s micro-abscess and inflammatory cell infiltration, particularly with T lymphocytes, macrophages and neutrophils, in the dermis and submucosa [128]. However, it should be remembered that histopathologic characteristics of both conditions may vary depending on clinical stage of the lesion and the biopsied area [48]. Despite the unknown etiology, some authors suggest that there is a genetic link between psoriasis and GT. One of the most well-known genetic factors of susceptibility for psoriasis is the human leukocyte antigen (HLA), located on the short arm of chromosome 6.46 [101]. Indeed, few studies demonstrated a link between GT and HLACw*06, the main allele of susceptibility to psoriasis [63, 128]. Also indirect proof for a common genetic background of GT and psoriasis exists: early onset psoriasis, often co-existing with GT, was shown to be more often associated with HLA-C*06 than the late-onset disease [9, 177]. Involvement of the same genetic factors in the etiopathogenesis of GT and psoriasis is also reinforced by the fact that 35% of patients with GT and psoriasis have a positive family history [176]. Aside from a potential link to HLA, also the presence of the polymorphism +3954 IL-1B was shown to be associated with increased risk of GT [68]; the product of the altered gene, interleukin 1B (IL-1B), is known to play a pivotal role in the pathogenesis of psoriasis as well [128]. Another argument for a pathogenic link between GT and psoriasis may be the fact that in one study, patients with the former condition presented with elevated salivary concentrations of tumor necrosis factor alpha (TNF-alpha) and interleukin 6 (IL-6), i.e., cytokines implicated in the pathogenesis of psoriasis [5].

Also environmental influences seem to play a role in the development of GT in psoriatics. GT is known to be linked to alcohol consumption and stress, and psoriatic patients are exposed to these two factors to a markedly larger extent than individuals from the general population [61]. Some studies demonstrated that GT develops less often in cigarette smokers due to smoking-related changes in the tongue epithelium: enhanced keratinization and a decrease in the synthesis of TNF-alpha, IL-1 and IL-6 by macrophages, associated with activation of nicotine receptors in these cells [26, 61, 77]. While the role of smoking as a factor protecting against the development of GT is still unestablished, it was already confirmed in other oral diseases, such as aphthous stomatitis [11, 163]. Considering that neutrophils play a key role in both psoriasis and aphthous stomatitis, perhaps morphological and functional changes in these cells caused by cigarette smoking [94] have a role in this protection mechanism. If this is true, a large proportion of smokers among psoriatics may at least partially explain why GT is found in only less than 20% of this group.

Fissured tongue is another non-specific oral pathology found in 6–47.5% of psoriatic patients [47, 131, 182]. FT, also referred to as lingua fissurata, lingua plicata, scrotal tongue or grooved tongue, is recognized clinically by an anteroposterior groove, often with multiple lateral fissures [182]. The frequency of FT increases with age [49, 74] and is usually higher among men [38, 43]. Epidemiological studies demonstrated that aside from psoriasis, FT is also found in patients with acromegaly, Sjögren’s, Down and Melkersson-Rosenthal syndromes [138].

Compared to GT, our knowledge about the link between FT and psoriasis is fairly limited. While GT is a transient lesion, FT seems to be a permanent pathology of the tongue [165]. Furthermore, a specific sequence, with the development of GT in early psoriasis and manifestation of FT at further stages of the disease, has been reported by some authors [129]. However, it appears that there is no significant difference in the occurrence of FT in early and late-onset psoriasis [182]. This may be explained, at least in part, by the fact that the incidence of this condition increases with age [182]. According to some authors, likewise GT, also FT is more common in generalized pustular psoriasis [37]. Despite familial occurrence of both GT and FT [62], no genetic background for the former pathology was found in psoriatic patients [34, 62]. Consequently, when associated with GT, FT seems to be a sequel of the latter [130].

In conclusion, the abovementioned data about the coexistence of psoriasis with non-specific oral lesions have some important clinical implications. First of all, thorough evaluation of oral mucosa should become a routine component of a dermatological examination. Whenever suspected lesions are found in the psoriatic with psoriasis, differential diagnosis should be conducted to exclude candidiasis, lichen planus, erythroplasia, systemic lupus erythematosus (SLE), trauma, drug reactions and other potential non-psoriatic etiologies [10]. If all these conditions are excluded based on clinical and whenever necessary, also histopathologic examination, modification of anti-psoriatic treatment may be considered, since a scarce evidence from individual case reports suggests that psoriatic patients with concomitant GT and/or FT may respond well to systemic treatment with retinoids or anti-TNF agents [24, 41].

### Inflammatory bowel disease

Inflammatory bowel disease (IBD), i.e., Crohn’s disease (CD) and ulcerative colitis (UC), develops due to inappropriate immune response to commensal microorganisms in genetically predisposed individuals [58, 141, 154, 168, 169]. A review of literature showed three potential epidemiologic links between IBD and psoriasis: (1) higher incidence of secondary psoriasis in patients with CD or UC, (2) predisposition to IBD among psoriatics, and (3) induction of iatrogenic psoriatic lesions in IBD patients treated with anti-TNF agents.

The first data on a potential link between the two conditions originate from 1968 when the prevalence of psoriasis in first-degree relatives of patients with CD was shown to be two to threefold higher than in the controls [70]. Further studies confirmed that individuals with IBD and their close relatives are predisposed to the development of psoriatic lesions to a markedly larger extent than subjects from the general population, and the incidence of psoriasis in the former group can be even up to fivefold higher [96]. This association was observed irrespective of the age at which the diagnosis of IBD was made, intestinal segment involved and patient sex [96]. Also an inverse phenomenon has been observed, i.e., higher incidence of IBD in psoriatics [30]. Available evidence suggests that the risk of CD and UC during the course of psoriasis is more than twofold and nearly twice higher than in the general population, respectively, also after exclusion of patients treated with anti-TNF agents [30]. Furthermore, some studies demonstrated that despite the lack of clinical abnormalities, psoriatic patients may present with microscopic evidence of intestinal inflammation and elevated levels of pANCA, i.e., the features of a latent IBD [87, 100, 166]. Indeed, there is a case report of a patient with psoriasis who developed clinical CD after up to 15 years since diagnosis of the primary condition. The authors of this report hypothesized that it was previous anti-psoriatic treatment which likely masked the clinical symptoms of IBD and contributed to the diagnostic delay [158].

**Fig. 3:**
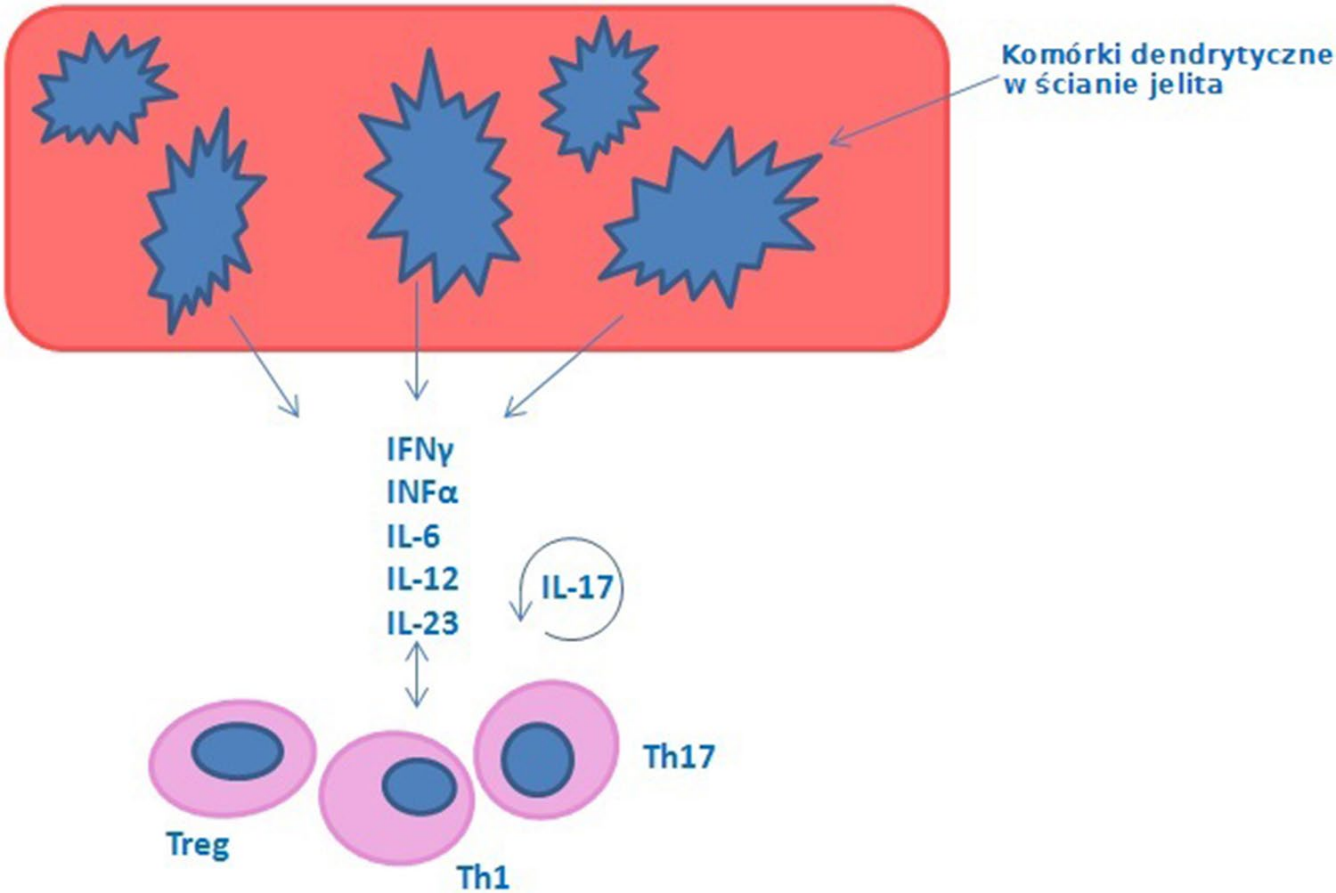
Mechanisms enhancing an increase of intestinal permeability in inflammatory bowel disease IBD involve several group of cells—dendritic cells “in situ in the intestinal wall”, T-regulatory lymphocytes (T-reg), Th17 cells, Th1 cells, which are responsible for producing cytokines such as IFNγ, INFα, Il-6, IL-12, IL-23 and IL-17. Based on Vlachos et al. [167]

The above-mentioned associations can be at least partially explained by a common genetic background of psoriasis and IBD. Several areas of chromosomes 16, 6, 4 and 3 were found to contain common genetic markers of psoriasis and IBD [22, 28, 45, 78, 79, 109, 119, 121, 145, 174]. All loci determining susceptibility to both conditions, among them the IBD3 locus involved in CD and UC, and PSORS1 locus involved in psoriasis, were found in the 6P21 region encompassing the major histocompatibility complex (MHC) [170]. Aside from the MHC components, also a few other genes, specifically those encoding interleukin 23 receptor (IL-23R) and interleukin 12B (IL-12B) were implicated in the pathogenesis of both psoriasis and IBD [21, 44]. Furthermore, both diseases share some common inflammatory pathways. Both psoriasis and IBD are Th1-mediated inflammatory disorders associated with enhanced synthesis of cytokines, TNF-alpha and interferon-gamma (IFN-gamma). Also Th17 cells, synthesizing interleukins-17 and -21 (IL-17 and IL-21), as well as IFN-gamma, play a pivotal role in the pathogenesis of both conditions [7, 156, 173]. Th17 cells promote acanthosis, hyperkeratosis, and parakeratosis, as well as the synthesis of inflammatory molecules within the dermis and epidermis [8, 50]. In psoriatic patients, biopsy specimens from injured skin showed a high number of Th17 [95, 142]. Aside from elevated serum concentrations of IL-17 and IL-23, increased levels of these cytokines were also found in the intestinal lamina propria of individuals with CD and in the skin lesions of psoriatic patients [42]. Also abnormalities in the number and function of T-regulatory lymphocytes (T-regs) have been described in both psoriasis and IBD [152] (Fig. 3). In the active phase of CD and UC, the number of T-regs in peripheral blood is lower than in the controls; this phenomenon is not observed during remission of these conditions, suggesting that in the course of IBD, T-regs migrate from peripheral blood to the inflamed intestinal mucosa [144, 179]. An altered recruitment and/or function of T-regs can be also an important pathogenic factor in skin diseases, including psoriasis, although the exact mechanisms are yet to be established [16, 76].

As already mentioned, there is also an evident iatrogenic link between IBD and psoriasis. A number of authors reported that some patients with IBD may develop psoriatic lesions during the course of anti-TNF treatment, with either etanercept or anti-TNF antibodies, such as infliximab (IFX) or adalimumab (ADA) [35, 67, 157]. This phenomenon is seemingly paradoxical, as anti-TNF agents are also effective in psoriatics [66, 111, 146]. Epidemiologic data suggest that this side effect is unlikely related to common genetic and pathogenic backgrounds of both conditions; a latency period between the development of secondary non-iatrogenic psoriasis is longer, usually up to a few years [157]. Although highly variable (ranging from days to even 4 years), mean time elapsed since the initiation of an anti-TNF treatment to the onset of psoriatic lesions was estimated at 10.5 months in a review of 127 reported cases [90]. Furthermore, development of the secondary psoriatic lesions was observed not only in patients receiving anti-TNF agents due to IBD, but also in individuals who received drugs from this group due to the presence of other immune-mediated conditions, such as rheumatoid arthritis (RA), ankylosing spondylitis and juvenile arthritis [32, 90, 171]. The incidence of psoriasis during anti-TNF treatment for various clinical conditions has been estimated at 0.6–5.3% [73, 90], and at 1.6–2% in patients who received anti-TNF agents due to IBD [13, 135]. A greater absolute frequency of psoriasis cases has been reported with the use of IFX than with ADA or etanercept in the treatment of IBD [67]. The onset of psoriatic lesions during anti-TNF treatment can follow three primary patterns: (1) psoriasiform eruption with typical histopathological features of a drug reaction, showing lichenoid or interface dermatitis, (2) exacerbation of preexisting psoriasis, and (3) *de novo* psoriasis [169]. When the skin lesions develop in patients with a history of psoriasis, they are usually found in a previously unaffected location and often have an atypical appearance [35].

Although Il-17 serum is elevated both in psoriasis and CD, while anti-TNFs work in both psoriasis and IBD, anti-IL17 works in psoriasis but not in IBD and may even exacerbate IBD. This strengthens the importance of the link between psoriasis and IBD [35, 51, 67].

According to most authors, there is an increase in interferon-alpha (IFN-alpha) level due to reduced concentration of circulating TNF-alpha, which leads to the development of psoriatic lesions [120, 124]. Indeed, some studies demonstrated that the secondary psoriatic lesions developed during the course of anti-TNF treatment contain higher levels of IFN-alpha than those associated with idiopathic psoriasis [40, 54]. Furthermore, treatment with IFN-alpha, either systemic or topical, was shown to exacerbate psoriasis [56, 93]. Other proposed underlying mechanisms of secondary psoriasis include anti-TNF treatment-induced activation of autoreactive T-cells [90, 147] or certain infectious agents such as *Streptococcus* spp. [12, 90, 97]. Interestingly, the incidence of palmoplantar pustulosis in patients treated with anti-TNF agents was shown to be at least twice as high as in the general population (40 vs. less than 20%) [90]. It has been suggested that this may be due to the high expression of TNF-alpha in the palmar eccrine sweat ducts [114]. There seems to be a genetic predisposition to secondary psoriasis as well, since the vast majority of patients receiving anti-TNF treatment do not develop skin lesions [35]. However, most patients with secondary psoriasis do not have a family history of this condition [82, 90, 135, 181]. While one study demonstrated that patients with anti-TNF treatment-associated secondary psoriasis had modestly greater genetic predisposition towards the latter condition, no single causative polymorphism was identified [167]. Also a contribution of an environmental factor cannot be excluded owing to the previously mentioned heterogeneity in the time elapsed since the initiation of anti-TNF treatment to the onset of psoriatic lesions.

Irrespective of the etiology thereof, the hereby presented associations between IBD and psoriasis have some important clinical implications. First, the hereby presented data suggest that individuals with CD or UC should be regularly followed up by a dermatologist and thoroughly examined for the presence of potential psoriatic lesions, especially during the course of anti-TNF treatment and thereafter. The same refers to patients with psoriasis, in whom information on potential GI ailments suggestive of IBD should be obtained during each control visit, and gastroenterological consultation should be sought whenever necessary. Available evidence suggests that patients who developed secondary psoriasis during the course of anti-TNF treatment for IBD should probably continue the therapy [67]. Experiences of other authors imply that discontinuance of anti-TNF agents may result in exacerbation of the primary condition, and topical treatment is sufficient to control secondary psoriasis in most cases [31]. However, discontinuation of the anti-TNF agent should be considered in patients who did not respond adequately to local treatment of psoriatic lesions. Such approach, with maintenance of either topical or systemic treatment for psoriasis (methotrexate, retinoids or cyclosporine), proved to be adequate in patients with severe psoriatic lesions covering more than 5% of the body surface area, and in individuals with pustulosis [31]. When secondary psoriatic lesions are not severe but negatively affect the patient’s quality of life, temporary suspension of the biological agent with subsequent reintroduction with strict clinical monitoring may be an option [67].

### Celiac disease

Celiac disease is an autoimmune condition triggered by ingestion of gluten in genetically predisposed individuals [137]. Aside from GI ailments, this systemic disease, affecting ca. 1% of the general population, may also predispose to the development of skin lesions, endocrine disorders, iron deficiency anemia, osteoporosis, hypertransaminasemia, neurological disorders and even cancer [141]. The presence of celiac disease-specific antibodies against gliadin, reticulin, glutaminase and smooth muscle endomysium was also reported in subjects with psoriasis and other autoimmune and inflammatory conditions, such as SLE, RA and Sjögren’s syndrome [23, 36, 83, 102, 113, 123, 136]. Importantly, the level of these antibodies was shown to correlate with the severity of psoriasis [172]. Aside from this serological evidence, also epidemiologic links between psoriasis and celiac disease have been documented [2, 14, 122]. According to various authors, 0.2–4.3% of psoriatics may present with concomitant celiac disease, and odds ratios for coexistence of these two conditions were consistently shown to be around 2 [2, 14, 106, 118, 122]. Importantly, a large nationwide study demonstrated that subjects with celiac disease are at increased risk of psoriasis both before and after the diagnosis of gluten intolerance [106]. However, epidemiologic evidence is still inconclusive, since some studies did not demonstrate an association between psoriasis and celiac disease, either at a clinical or serological level [33, 89]. Furthermore, several studies that documented such a link suffered from limitations including small numbers of patients with celiac disease and lack of a control group [162].

If it truly exists, the association between celiac disease and psoriasis may be explained by several mechanisms. First, malabsorption associated with celiac disease may predispose to vitamin D deficiency [15]; also gluten-free diet used in the treatment of celiac disease is often deficient, regarding in this vitamin [92]. Vitamin D deficiency is known to predispose to psoriasis, and exposure to sun light and administration of topical vitamin D analogs in creams produced beneficial effects in psoriatic patients [98]. Second, although celiac disease is generally associated with Th2 response, also Th1 and Th17 cells [29, 99, 143], i.e., the lymphocyte subpopulations involved in the development of psoriasis, play an important role in the pathogenesis of this condition [91]. Recently, Skavland et al. [151] demonstrated that some wheat antigens may trigger an immune response in psoriatic patients significantly more often than in non-psoriatic controls, inducing expression of cutaneous lymphocyte antigen (CLA). Third, also a common genetic background may explain the link between psoriasis and celiac disease. Genome-wide association studies of these two conditions identified genetic susceptibility loci at eight genes regulating innate and adaptive immune response: TNFAIP3, RUNX3, ELMO1, ZMIZ1, ETS1, SH2B3, SOCS1 and UBE2L3 [105, 159, 161]. The fourth implicated mechanism may be associated with an increase in the intestinal permeability [3], a characteristic feature of celiac disease that has been also found in some psoriatics [80] (Table 3).

**Table 3:** Links between psoriasis and celiac disease

Association between celiac disease and psoriasis
Vitamin D deficiency
Th2 response, also Th1 and Th17 cells [expression of cutaneous lymphocyte antigen (CLA)]
Genetic background
Increase in the intestinal permeability

Although available data regarding coexistence of celiac disease and psoriasis are still inconclusive and potential shared etiopathogenic mechanisms remain mostly hypothetical, a large body of evidence suggests that psoriatic patients, either with concomitant celiac disease or asymptomatic gluten intolerance, may benefit from gluten-free diet. In a study of psoriatic patients who tested positively for anti-gliadin antibodies (AGA), 3-month gluten-free diet resulted in a significant decrease in the affected area of the skin, PASI scores and AGA titers [113]. Interestingly, ca. 50% of the AGA-positive patients did not show endoscopic evidence of celiac disease prior to implementation of the gluten-free diet, which suggests that such diet may be also beneficial in psoriatics with asymptomatic gluten sensitivity [25]. Also in another study, implementation of a gluten-free diet resulted in a decrease in tissue transglutaminase expression in AGA-positive patients with psoriasis [112]. Finally, a few case reports documented complete resolution of skin lesions after administration of gluten-free diet to psoriatics with serological evidence of gluten intolerance [4, 39, 53, 69]. Altogether, these findings suggest that gluten-free diet may produce beneficial effects in most psoriatic patients who tested positively for celiac disease-specific antibodies.

To summarize, relatively frequent coexistence of celiac disease and psoriasis justifies monitoring of patients with either condition for clinical evidence of the other. Furthermore, serum levels of vitamin D should be regularly controlled in patients with celiac disease, either with concomitant psoriasis or without. Even more importantly, implementation of gluten-free diet should be considered in psoriatics presenting with serological evidence of gluten intolerance or clinical signs of celiac disease.

### Non-alcoholic fatty liver disease

Non-alcoholic fatty liver disease (NAFLD) is a heterogeneous condition including both relatively benign simple fatty liver and severe non-alcoholic steatohepatitis, which may eventually result in fibrosis and cirrhosis and give rise to hepatocarcinoma [58]. NAFLD is diagnosed in 20–30% of individuals from the general population, and represents an established cardiovascular risk factor and common manifestation of the metabolic syndrome also usually coexists with insulin resistance [104, 155].

Owing to the frequent occurrence of the metabolic syndrome in psoriatic patients, high incidence of NAFLD in this group is not surprising. Available evidence suggests that the risk of NAFLD in psoriatics is approximately twice as high as in the general population (48–59%) [58, 60, 115]. Importantly, this association seems to occur independently of the administration of potentially hepatotoxic anti-psoriatic medications, such as methotrexate and anti-TNF agents [60]. Furthermore, the results of several studies imply that whenever they coexist in the same patient, psoriasis and NAFLD may perpetuate the course of each other. The presence of NAFLD was shown to be associated with greater severity of psoriasis and a higher risk of joint involvement. In turn, individuals with NAFLD and concomitant psoriasis were more prone to the development of liver fibrosis than non-psoriatic controls [115].

The etiopathogenic link between psoriasis and NAFLD is not straightforward, as recently both these entities have been increasingly recognized as systemic conditions. Some pro-inflammatory cytokines synthesized by lymphocytes and keratinocytes in psoriatic skin, including IL-6, IL-17 and TNF-alpha, may contribute to systemic insulin resistance [148, 164], a common feature of NAFLD. Another important contributor is inflamed visceral adipose tissue (VAT) that perpetuates both chronic inflammation and liver damage due to enhanced secretion of various factors, such as non-esterified fatty acids, hormones and pro-inflammatory adipokines (TNF-alpha, IL-6, visfatin, leptin and resistin), as well as due to decreased production of adiponectin [19, 20, 81, 103, 149]. As a result, the liver of obese and/or insulin-resistant individuals is exposed to high plasma concentrations of non-esterified fatty acids, being a principal factor of oxidative and cytokine-induced liver damage [19, 20, 81, 103, 149]. Although this relationship has not been yet confirmed experimentally, enhanced release of non-esterified fatty acids from inflamed VAT may also contribute to the development of psoriasis [107]. On the other hand, NAFLD, especially its more severe forms, may aggravate insulin resistance, predispose to dyslipidemia and enhance synthesis of pro-inflammatory, pro-coagulant, pro-oxidant and pro-fibrogenic mediators (CRP, IL-6, fibrinogen, plasminogen activator inhibitor 1 and tumor growth factor beta) in the liver [6, 19, 20, 153]. All these factors may be involved in the pathogenesis of psoriasis, stimulating proliferation of keratinocytes, skin inflammation and synthesis of various adhesion molecules [107]. Also the role of environmental and iatrogenic factors should be considered in this vicious circle. For example, cigarette smoking and alcohol consumption, both common among psoriatics, may predispose to NAFLD and enhance liver fibrosis, as well as directly interfere with the course of psoriasis [1]. In turn, many systemic anti-psoriatic medications, especially methotrexate, may contribute to iatrogenic damage of hepatocytes, liver steatosis and fibrosis, whereas long-term administration of steroids is an established risk factor of insulin resistance, diabetes mellitus, obesity and hyperlipidemia [46, 57, 59, 72, 88, 139].

The unquestioned link between psoriasis and metabolic syndrome, including its liver component, NAFLD, has important clinical implications. First, all psoriatic patients should be regularly screened for all components of the metabolic syndrome and encouraged to lifestyle modifications, such as dietary changes, weight control and greater involvement in physical activity. Secondly, liver function should be thoroughly monitored during the course of anti-psoriatic treatment, and the therapeutic protocol should be modified appropriately whenever any evidence of impaired hepatic function emerged.

### Psoriasis and cancer

The chronic and immune-mediated character of the primary condition, iatrogenic factors (long-term administration of immunosuppressive agents, phototherapy) and greater exposure to some established environmental carcinogens (cigarette smoke, alcohol) may predispose psoriatic patients to carcinogenesis, also within the GI tract [132]. Indeed, several epidemiologic studies including one large meta-analysis, demonstrated that psoriasis is associated with increased evidence of cancer overall [17, 27, 52, 108, 134], and may predispose to the development of GI malignancies, specifically oral, esophageal, liver and pancreatic cancer [17, 52, 71, 86, 126, 160]. However, psoriasis was no longer associated with the excess cancer risk whenever the results were adjusted for cigarette smoking and alcohol consumption data, if available [17, 134].

Based on available evidence, psoriatic patients may be at an increased risk of carcinogenesis within the GI tract, but the excess incidence of cancers in this location does not seem to be related to the biology of the primary condition or treatment thereof, but rather to the negative impact of skin lesions on lifestyle. However, it cannot be excluded that the increased risk of carcinogenesis in psoriatics may be also attributed to concomitant GI pathologies presented in this review, specifically IBD, NAFLD and celiac disease, since all of them are known to predispose to cancer. However, to the best of our knowledge, this association has not been studied thus far. Nevertheless, individual risk of cancer should be estimated for each psoriatic patient, taking into account both general risk factors, such as age, family history, environmental and occupational exposures, and disease-specific factors, such as anti-psoriatic treatments and comorbidities.

## Discussion

1. Published evidence summarized in this review suggests that psoriasis is considered as a systemic condition and may co-exist with numerous GI pathologies, especially those with established immune-related mechanisms.
2. Predisposition to some diseases of the GI may be associated with anti-psoriatic treatment.
3. It seems that psoriatic patients may be at a high risk of carcinogenesis within digestive tract but this issue requires further researches.
